# The Step Further to Understand the Role of Cytosolic Phospholipase A_**2**_ Alpha and Group X Secretory Phospholipase A_**2**_ in Allergic Inflammation: Pilot Study

**DOI:** 10.1155/2014/670814

**Published:** 2014-08-27

**Authors:** Ewa Pniewska, Milena Sokolowska, Izabela Kupryś-Lipińska, Monika Przybek, Piotr Kuna, Rafal Pawliczak

**Affiliations:** ^1^Department of Immunopathology, Faculty of Biomedical Sciences and Postgraduate Training, Medical University of Lodz, 7/9, Zeligowskiego Street, 90-752 Lodz, Poland; ^2^Critical Care Medicine Department, Critical Center, National Institutes of Health, Bethesda, MD 20892-1662, USA; ^3^Department of Internal Diseases, Asthma and Allergy, Medical University of Lodz, 90-153 Lodz, Poland

## Abstract

Allergens, viral, and bacterial infections are responsible for asthma exacerbations that occur with progression of airway inflammation. cPLA_2_ *α* and sPLA_2_X are responsible for delivery of arachidonic acid for production of eicosanoids—one of the key mediators of airway inflammation. However, cPLA_2_ *α* and sPLA_2_X role in allergic inflammation has not been fully elucidated. The aim of this study was to analyze the influence of rDer p1 and rFel d1 and lipopolysaccharide (LPS) on cPLA_2_ *α* expression and sPLA_2_X secretion in PBMC of asthmatics and in A549 cell line. PBMC isolated from 14 subjects, as well as A549 cells, were stimulated with rDer p1, rFel d1, and LPS. Immunoblotting technique was used to study the changes in cPLA_2_ *α* protein expression and ELISA was used to analyze the release of sPLA_2_X. PBMC of asthmatics released more sPLA_2_X than those from healthy controls in the steady state. rDer p1 induced more sPLA_2_X secretion than cPLA_2_ *α* protein expression. rFel d1 caused decrease in cPLA_2_ *α* relative expression in PBMC of asthmatics and in A549 cells. Summarizing, Der p1 and Fel d1 involve phospholipase A_2_ enzymes in their action. sPLA_2_X seems to be one of important PLA_2_ isoform in allergic inflammation, especially caused by house dust mite allergens.

## 1. Introduction

Despite intensive studies on asthma pathogenesis and seeking the effective treatment, the number of new cases is increasing globally. Asthma is a very heterogeneous disease whose etiology has not been fully understood. Allergens, drugs, viral and bacterial infections, and stress are the most common factors that initiate and exacerbate asthma. About 70% of asthmatics are atopic [1]. The proposed mechanism of allergic asthma development suggests that allergen exposure causes sensitization, and continued exposure leads to airway hyperresponsiveness and inflammation. Airway inflammation primarily initiated as a defense process aiming to eliminate the damaging factor, which evolves to chronic state causing airway remodeling and impaired lung functions. In general population of Lodz province (Poland) the most common sensitizing indoor allergens are house dust mites and cat [2]. Sensitivity to house dust mite and cat dander are risk factors associated with the development of asthma [3]. Many studies indicate that allergen exposure causes the exacerbation of asthma that occurs with impaired lung function and increases the need for hospitalization [4, 5].

Group X secretory phospholipase A_2_ (sPLA_2_X) has recently been investigated as one of the most important members of secretory PLA_2_ in the inflammatory process [6]. Except its enzymatic activity sPLA_2_ can act through the membrane receptors causing cell degranulation and initiating chemokines and cytokines production [7, 8]. Moreover, sPLA_2_ can influence cytosolic PLA_2_ (cPLA_2_) action [9, 10]. In human airways a lot of resident cells (mast cells, macrophages, endothelial cells, epithelial cells, and bronchial smooth muscle cells (SMC)) and haematopoetic cells (basophils, eosinophils, neurophils, lymphocytes, and monocytes) are potential source of secretory phospholipases [11]. In asthmatics expression of sPLA_2_-X predominates in airway epithelium. Moreover, both sPLA_2_-X and sPLA_2_-IIA are the main phospholipases detected in BAL fluid [6, 12]. sPLA_2_-X and sPLA_2_-XII are elevated in induced sputum cells of patients with asthma [13]. The studies with knockout mice showed that deficiency of sPLA_2_-X reduced allergen-induced features of airway inflammation [14].

Cytosolic phospholipase A_2_ group IVA (cPLA_2_ *α*) is the most potent enzyme in phospholipase A_2_ superfamily catalyzing liberation of arachidonic acid (AA) from membrane phospholipids [15]. Our previous studies revealed that cPLA_2_ *α* participates in asthma pathogenesis [16]. What is more, rDer p1 caused overexpression of* PLA2G4A* in PBMC of asthmatics (unpublished data). Whalen et al. showed that PBMC of asthmatic patients stimulated with allergens in the presence of cPLA_2_ inhibitor exhibited decreased production of proinflammatory cytokines [17]. cPLA_2_ actions are mainly regulated by Ca^2+^ concentration and serine residue phosphorylation [18, 19]. LPS can modulate activity of cPLA_2_ by phosphorylation [20]. Der p1 can activate MAPKs in different types of cells [21, 22]. Despite the abovementioned facts that prove the Der p1-cPLA_2_ *α* interactions, other mechanisms of allergens impact on lipid mediators remains not fully understood. Thus, we investigated whether allergens or LPS can directly stimulate the expression and/or phosphorylation of cPLA_2_ protein in PBMC of severe asthmatics with atopic origin.

## 2. Material

### 2.1. Patients

Patients (*n* = 7) with severe asthma, who were allergic to house dust mite (Der p1) and cat (Fel d1) allergens, and healthy controls (*n* = 7) were enrolled to the study. The project was approved by the local ethics committee and an informed consent was obtained from every subject prior to the study. Patients were recruited from the Department of Internal Diseases, Asthma and Allergy of Medical University of Lodz. Asthma was recognized at least 6 months prior to the study and met the criteria of GINA Guidelines [23]. The severity of the disease was assessed according to the American Thoracic Society Workshop on Refractory Asthma 2000 Report [24]. All patients were classified as severe asthmatics. Patients were asked to not administer antihistamine drugs, oral gluccocorticoids and leukotriene receptors antagonists 24 hours, and inhaled glucocorticoids and long-acting beta agonists 12 hours before blood drawing. Healthy volunteers had no known history of asthma or seasonal allergies.  Table 1 presents characteristics of subjects enrolled to the study.

### 2.2. PBMC

Peripheral blood mononuclear cells (PBMC) were isolated using Histopaque 1077 (Sigma-Aldrich, St. Louis, MO), the density gradient cell separation medium according to the producer's instructions. Cells were cultured in RPMI1640 (Sigma-Aldrich, St. Louis, MO) with 10% heat-inactivated FBS (Sigma-Aldrich, St. Louis, MO) and antibiotics. 10 ng/mL of polymyxin B was added to medium used in allergen stimulation and 100 U/mL penicillin and 100 *μ*g/mL streptomycin (Sigma-Aldrich, St. Louis, MO) for LPS incubation. 2 × 10^6^/mL PBMC were stimulated* in vitro *with LoTox deglycosylated recombinant* Dermatophagoides pteronyssinus *allergen 1 (rDer p1), LoTox deglycosylated recombinant* Felis domesticus* allergen 1 (rFel d1) (Indoor Biotechnologies, Cardiff, UK) or LPS from* E. coli*, serotype R515 (Enzo Life Sciences, NY). In dose-response systems three concentrations of allergens: 1 *μ*g/mL, 5 *μ*g/mL, and 10 *μ*g/mL and LPS: 50 ng/mL, 100 ng/mL, and 500 ng/mL were tested (at 24 h). In time-course system 5 *μ*g/mL of each allergen and 100 ng/mL of LPS were used and cells were collected in various time points: 0.5 h, 1 h, 2 h, 6 h, and 24 h.

### 2.3. A549 Culture

A549 cells, a human adenocarcinoma cell line, were obtained from the European Collection of Cell Cultures, Heath Protection Agency (Salisbury, UK) and were grown in Ham's F-12 K medium (Sigma-Aldrich, St. Louis, MO) with 10% fetal bovine serum (Sigma-Aldrich, St. Louis, MO), 2 mM of L-glutamine (Sigma-Aldrich, St. Louis, MO), 100 unit/mL penicillin, and 100 *μ*g/mL streptomycin (Sigma-Aldrich, St. Louis, MO). All experiments were performed when cells were 80% to 90% confluent. Cells were stimulated* in vitro* with LoTox deglycosylated recombinant* Dermatophagoides pteronyssinus *allergen 1 (rDer p1), LoTox deglycosylated recombinant* Felis domesticus* allergen 1 (rFel d1) (Indoor Biotechnologies, Cardiff, UK), or LPS from* E. coli*, serotype R515 (Enzo Life Sciences, NY).

## 3. Methods

### 3.1. Immunoblotting

Total protein from PBMC of patients with asthma, healthy subjects, and A549 cells was extracted in RIPA protein extraction buffer (Sigma-Aldrich, St. Louis, MO), supplemented with protease inhibitor cocktail (Sigma-Aldrich, St. Louis, MO). The lysate was centrifuged at 14,000 RPM and 4°C for 20 min, and the pellet discarded. Protein concentrations were determined by the BCA Protein Assay Kit (Pierce Thermo Scientific, Rockford, IL) according to the manufacturer's protocol and using bovine serum albumin as a standard. 20 *μ*g of total protein was mixed with NuPAGE LDS Sample Buffer (Life Technologies, Carlsbad, CA) and in a 1 : 10 ratio with NuPAGE Reducing Agent (10x), heated for 10 min at 70°C. Protein samples were subjected to electrophoresis in 4–12% SDS-NuPAGE Gels (Life Technologies, Carlsbad, CA) at 200 V and electrophoretically transferred to a nitrocellulose membrane at 30 V for one hour. The membrane was blocked in 5% nonfat milk in TBST (20 mM Tris-HCL, 500 mM NaCl, 0.05% Tween 20, and pH 7.5) for 1 hour at room temperature. Then, the membranes were incubated for 12 h at 4°C with the one of the following antibody: polyclonal rabbit anti-cPLA_2_ and anti-phospho-cPLA_2_ (Ser505) and anti-*β*-actin antibodies (Cell Signaling, Danvers, MA). At the end of the overnight incubation, the membrane was washed with TBST and incubated for one hour in TBST containing the goat anti-rabbit IgG secondary antibodies conjugated with alkaline phosphatase (Sigma-Aldrich, St. Louis, MO). After incubation with secondary antibodies, the membrane was washed three times (3 × 5 mins) in TBST buffer. The band was developed using BCIP/NBT alkaline phosphatase substrate (Merck Millipore, Darmstadt, Germany). Densitometric analysis of bands was performed with Image J 1.34s software (Wayne Rasband, National Institutes of Health, Bethesda, MD) and the results are presented as fold change of optical density (OD).

### 3.2. ELISA

The sPLA_2_X protein in supernatants form PBMC and A549 cells was measured by enzyme-linked immunoabsorbent assay (ELISA) using commercially available kit (Cloud-Clone Corp., Houston, TX) according to the manufacturer's protocol. The limit of detection for sPLA_2_X protein was 7.813 pg/mL.

### 3.3. Statistical Analysis

The data were analyzed using Statistica (v. 10.0; StatSoft, Tulsa, OK). Comparisons between groups were performed by using Mann-Whitney* U* tests or ANOVA followed by the Tukey's post hoc test when appropriate. Values of *P* < 0.05 were considered statistically significant.

## 4. Results

### 4.1. PBMC of Asthmatics Overproduced sPLA_2_X in the Steady State

**Figure 1:**
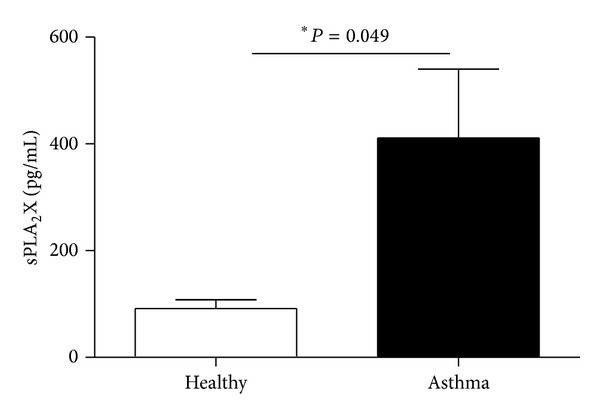
Concentration of sPLA_2_X released by PBMC of asthmatics and healthy controls. 2 × 10^6^ PBMC were incubated in medium containing RPMI1640 with 10% heat-inactivated FBS and antibiotics (37°C, 5% CO_2_). Supernatants were collected after 24 hours of incubation in 37°C. The secretion of sPLA_2_X was measured in indirect ELISA. Data are presented as mean ± SEM; ∗*P* < 0.05 as comparison between studied groups.

Hallstrand et al. [13] reported that sputum cells from asthmatics contained more sPLA_2_X mRNA (qPCR) and protein (immunostaining) in comparison to controls, so we hypothesized that similar observation in PBMC is very possible. Taking into account the fact that sPLA_2_X is produced as a zymogen and its cellular amount may not be relevant to its biological function we concluded that secretion of sPLA_2_X will be the best approach to discover its potential involvement in asthma pathogenesis. The levels of sPLA_2_X released by PBMC were compared in asthmatics and controls. The steady state concentration of sPLA_2_X was significantly higher in asthmatics (411.09 pg/mL ± 129.2) than in healthy subjects (91.96 pg/mL ± 16.37) (Figure 1).

### 4.2. rDer p1 Stimulation Results in Different sPLA_2_X and cPLA_2_ Production Patterns in Asthmatics and Controls

**Figure 2:**
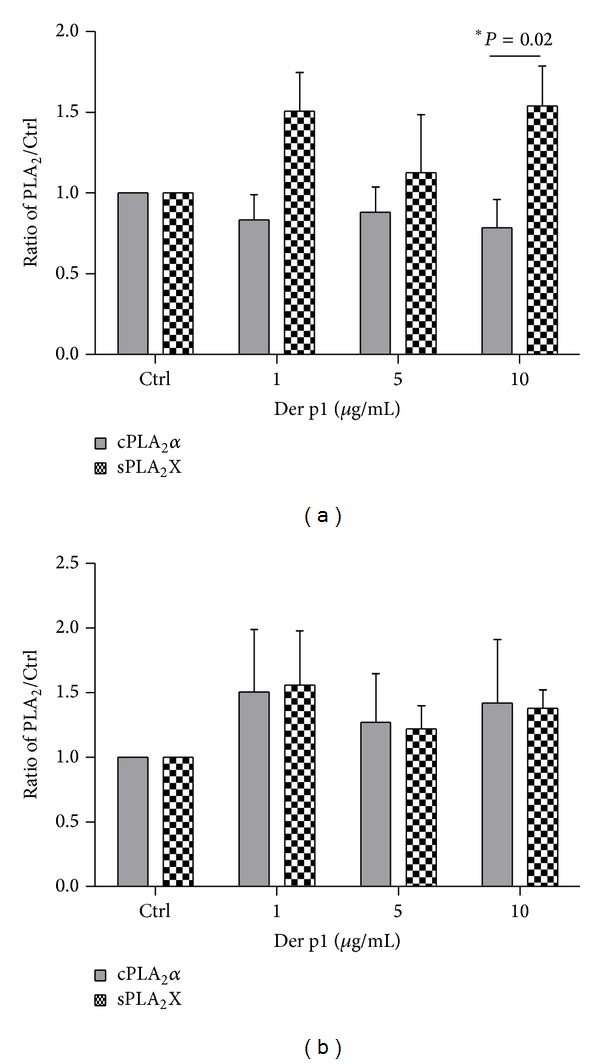
sPLA_2_X secretion and relative expression of cPLA*α* in PBMC of asthmatics (a) and healthy subjects (b) in response to rDer p1. PBMC (2 × 10^6^) were stimulated with indicated doses of rDer p1 for 24 hours.* Control *represents cells treated with the vehicle. The* bar graph *shows the densitometry results for cPLA_2 _(immunoblotting results) and ELISA results for sPLA_2_X secretion. Data are presented as the fold change compared with the vehicle-treated cells. Data represent the mean ± SE from at least six independent experiments. ∗*P *< 0.05 shows comparison between relative protein expression of cPLA_2_ and secretion of sPLA_2_X.

PBMC of asthmatics and healthy subjects were stimulated with rDer p1 for 24 hours in three different concentrations: 1, 5, and 10 *μ*g/mL. There were no differences in sPLA_2_X secretion between asthmatics and controls in any dose of rDer p1 whereas dust mite allergen in concentration of 10 *μ*g/mL significantly induced release of sPLA_2_X (1.54 ± 0.24) when compared with relative protein expression of cPLA_2_ *α* (0.79 ± 0.17) in asthmatics (Figure 2(a)). On the contrary, in healthy subjects, we did not observe similar fluctuation (Figure 2(b)).

### 4.3. Regulation of Relative cPLA_2_ Protein Expression by LPS, rFel d1, and rDer p1 in PBMC in Time-Dependent Manner

**Figure 3:**
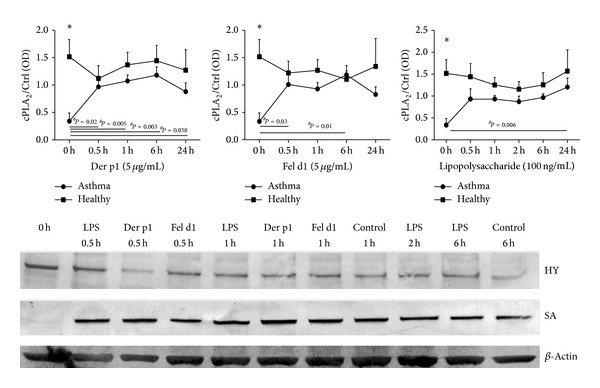
Relative cPLA_2_ *α* protein expression in PBMC from healthy subjects and asthmatic patients* in vitro* stimulated with rDer p1, rFel d1, and LPS in time-dependent manner. PBMC (2 × 10^6^) were stimulated with 100 ng/mL LPS or rDer p1 (5 *μ*g/mL) or rFel d1 (5 *μ*g/mL) at the indicated time.* Control *represents cells treated with the vehicle. The immunoblot is representative of experiments in PBMC from at least six donors, each showing similar results. The* line graph *shows the densitometry results. Data are presented as the fold change compared with the vehicle-treated cells. The point “0” indicates cPLA_2_ content in PBMC freshly isolated from blood, without culturing (untreated PBMC). Data represent the mean ± SE from at least six independent experiments. ^#^ *P* < 0.05**  **shows comparison with untreated cells; ∗*P* < 0.05  shows comparison between asthmatics and healthy subjects. HY: healthy subjects. SA: severe asthmatics.

**Figure 4:**
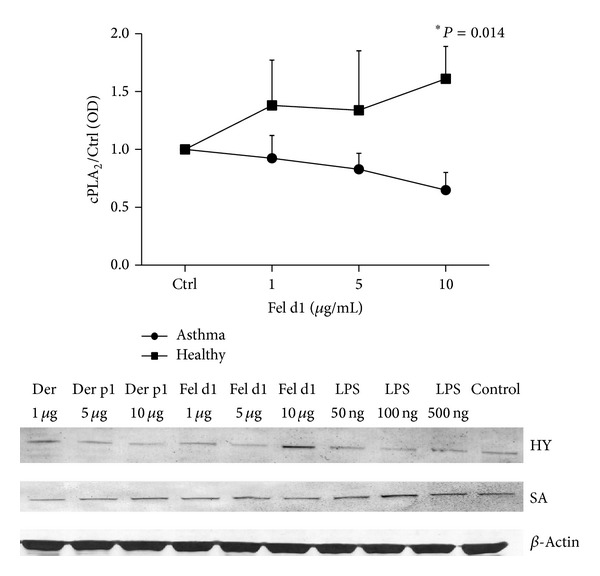
Relative cPLA_2_ *α* protein expression in PBMC from healthy subjects and asthmatic patients* in vitro* stimulated with rDer p1, rFel d1, and LPS in dose-dependent manner. PBMC (2 × 10^6^) were stimulated with indicated doses of LPS or rDer p1 or rFel d1 for 24 hours.* Control* represents cells treated with the vehicle. The immunoblot is representative of experiments in PBMC from at least six donors, each showing similar results. The* line graph* shows the densitometry results obtained from cells stimulated with rFel d1. Data are presented as the fold change compared with the vehicle-treated cells (control). Data represent the mean ± SE from at least six independent experiments. ∗*P* < 0.05shows comparison between studied groups. HY: healthy subjects. SA: severe asthmatics.

Relative expression of cPLA_2_ *α* protein was compared between healthy and asthmatic patients after stimulation with rDer p1 (5 *μ*g/mL), rFel d1 (5 *μ*g/mL), and LPS (100 ng/mL). cPLA_2_ *α* basal expression was significantly lower in asthmatics as compared to healthy subjects. While we did not observe differences in cPLA_2_ *α* protein synthesis between patients and controls after stimulation, there was a statistically significant increase of cPLA_2_ *α* protein expression in PBMC of severe asthmatics in all tested time points after stimulation with rDer p1 when compared to steady state level. Also rFel d1 induced expression of cPLA_2_ *α* protein after 0.5 h and 6 h of stimulation in patients. 24-hour incubation with LPS results in induction of cPLA_2_ in asthmatics when compared with level before stimulation. In healthy subjects cPLA_2_ *α* protein expression did not change significantly over time (Figures 3 and 4—blot).

### 4.4. cPLA_2_ Protein Synthesis Is Diminished by rFel d1 in PBMC of Asthmatics

PBMC from asthmatics and healthy subjects were stimulated with rDer p1, rFel d1, and LPS in three different concentrations for 24 hours. While being not significant (as compared to control), there was a trend of increased relative expression of cPLA_2_ in healthy subjects and decreased protein content in asthmatics after stimulation with rFel d1. However PBMC from asthmatics produced significantly less cPLA_2_ *α* (0.65 ± 0.15) than those from healthy subjects (1.61 ± 0.28) after stimulation with rFel d1 in concentration of 10 *μ*g/mL (Figure 4).

### 4.5. LPS Induced cPLA_2_ Phosphorylation in PBMC of Healthy Subjects

**Figure 5:**
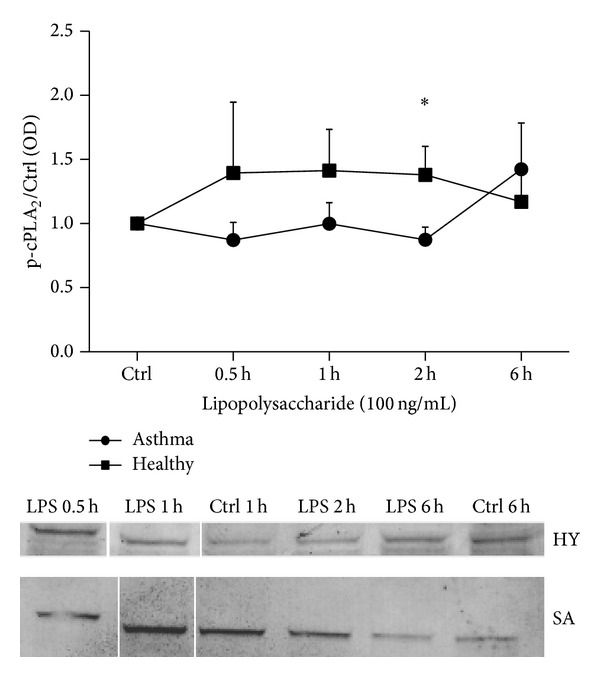
Phosphorylation of cPLA_2_ *α* protein in PBMC from healthy subjects and asthmatic patients* in vitro* stimulated with LPS. PBMC (2 × 10^6^) were stimulated with 100 ng/mL of LPS at indicated timepoints.* Control* represents cells treated with the vehicle. The immunoblot is representative of experiments in PBMC from at least six donors, each showing similar results. The* line graph *shows the densitometry results obtained from PBMC stimulated with LPS. Dataare presented as the fold change compared with the vehicle-treated cells. Data represent the mean ± SE from at least six independent experiments. ∗*P* < 0.05  shows comparison between studied groups.

Phosphorylation of cPLA_2_ *α* was analyzed in PBMC stimulated with rDer p1 (5 *μ*g/mL), rFel d1 (5 *μ*g/mL), and LPS (100 ng/mL). The cells from controls (1.38 ± 0.22) contained more phosphorylated form of cPLA_2_ *α* than patients' PBMC (0.87 ± 0.1) after 2 h of incubation with LPS. The rapid change in phosphorylation of cPLA_2_ *α* was observed after 6 h stimulation with LPS (Figure 5). The allergens did not change the phosphorylation of cPLA_2_.

### 4.6. Regulation of cPLA_2_ Protein Synthesis by LPS, rFel d1, and rDer p1 in A549 Cells

**Figure 6:**
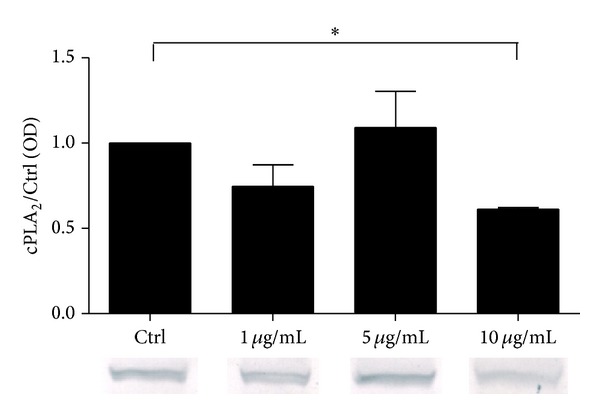
Relative cPLA_2_ *α* protein expression in A549 cells stimulated with rFel d1. A549 were stimulated with rFel d1 (5 *μ*g/mL) for 24 hours.* Control *represents cells treated with the vehicle. The immunoblot is representative of three independent experiments in A549 cells, each showing similar results. The* bar graph *shows the densitometry results. Data are presented as the fold change compared with the vehicle-treated cells. Data represent the mean ± SE from at least three independent experiments. ∗*P* < 0.05  shows comparison with untreated cells.

In A549 culture the rFel d1 in concentration 10 *μ*g/mL significantly decreased synthesis of cPLA_2_ (0.61 ± 0.01) (Figure 6). Any other stimulators did not change cPLA_2_ expression in short time of incubation.

### 4.7. Recombinant Der p1 Induces Morphological Changes in A549 Cells

**Figure 7:**
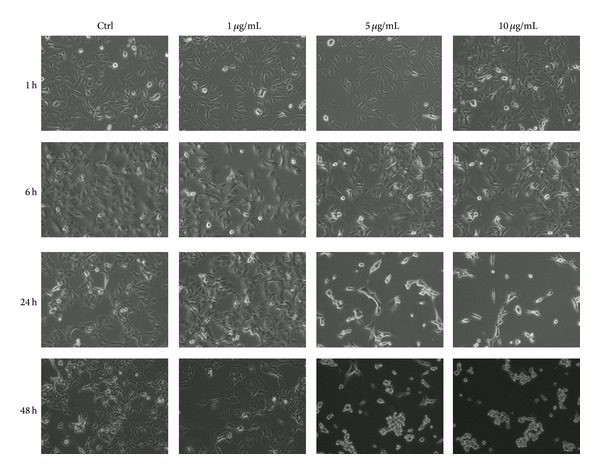
Morphological changes of A549 cells caused by rDerp1.

rDer p1 dose- and time-dependently caused morphological changes in A549 cells. Low concentrations and short incubation did not induce visible changes whereas higher concentrations and longer incubations led to cells shrinking and desquamation (Figure 7).

## 5. Discussion

We observed that asthmatics' PBMC released more sPLA_2_X than control cells in the steady state. This observation is supported by previous reports, showing that asthmatics have increased content of sPLA_2_X in airway epithelium, BALF, and sputum. The novelty of our study relates to regulation of different PLA_2_ isoforms in response to rDer p1 stimulation. Interestingly rDer p1 in highest dose (10 *μ*g/mL) significantly upregulated release of sPLA_2_X protein when compared to relative cPLA_2_ *α* protein expression in asthmatics whereas in healthy subjects we did not observe this tendency. This observation suggests that sPLA_2_X may be one of the important isoforms of PLA_2_ in allergic response. Despite evidence that both enzymes cooperate in liberation of AA, sPLA_2_X is also able to release AA independently to cPLA_2_ *α* [25] and therefore may alone promote allergen-induced inflammation. Misso et al. suggested that increased activity of sPLA_2_ may be associated with atopic status [26]. Some data related to the role of sPLA_2_X in airway inflammation come also from animal studies. Knock-in of human sPLA_2_X to msPLA_2_X^−/−^ mice restored allergen-induced inflammatory cell recruitment into airways as well as hyperresponsiveness to methacholine [27].

PBMC stimulated with rDer p1, rFel d1, or LPS showed increased production of cPLA_2_ *α* in comparison to steady state level in asthmatics but not in healthy subjects. The most rapid changes were observed after rDer p1 action, whereas 24-hour incubation with LPS was needed to induce significant increase of cPLA_2_ *α* content. The mechanism of Der p1 action is still not fully determined. Der p1 acts by PAR-2 receptor as well as in PAR-2-independent manner through activation of NF-*κ*B and ERK1/2 [22, 28]. Activation of NF-*κ*B pathway is prerequisite for cPLA_2_ expression in many cell types [29–31], so this pathway can partially be involved in the induction of cPLA_2_ *α* expression. In our study we did not observe the changes in production of cPLA_2_ *α* between asthmatics and controls after stimulation with allergens and LPS (time-response scheme).

Dose-response scheme of our experiment showed that rFel d1 in highest dose (10 *μ*g/mL) significantly decreased expression of cPLA_2_ *α* in asthmatics when compared to healthy subjects. The similar significant decrease of cPLA_2_ synthesis was observed in A549 cells after stimulation with rFel d1. Fel d1 has been shown to may have enzymatic activity [32]. The structural analysis of Fel d1 revealed homology of the allergen with *α*-subunit of mouse salivary androgen binding protein [33], uteroglobin and with the related Clara cell phospholipid binding protein, CC10 [34]. Uteroglobin is anti-inflammatory protein and can inhibit PLA_2_ activity [35]. The similarity between rFel d1 and uteroglobin may suggest that the allergen has cytokine-like properties; thus it may be capable of inflecting the immune response [34].

Although Der p1 and Fel d1 are able to increase activity and phosphorylation of cPLA_2_ in eosinophils [36], in our study we did not observe the significant changes in cPLA_2_ *α* phosphorylation after allergen stimulation. Only stimulation with LPS resulted in elevated level of phosphorylated cPLA_2_ *α* form in healthy subjects. PBMC of healthy volunteers respond better to LPS treatment. The diverse effects of LPS action have been observed earlier [37, 38]. Moreover it has been proved that LPS-induced cPLA_2_ activity is TLR-4-dependent [39]. Different experiment systems and doses of LPS used in experiments seem to condition the results of LPS stimulation. In U937 cell line and macrophages LPS significantly increased the expression of cPLA_2_ protein after 8 hours but not after 24 hours of stimulation whereas in tracheal smooth muscle cells the effective time points were 16 and 24 hours [30, 40].

rDer p1 in higher doses induces the desquamation of A549 cells. This effect was observed earlier and is result of enzymatic activity of the allergen [28]. Der p1 is a cysteine protease able to degrade the occludin protein and ZO-1 protein in tight junction between epithelial cells [28, 41].

## 6. Conclusions

Results of the study showed that Der p1 and Fel d1 involve phospholipase A_2_ enzymes in their action. sPLA_2_X seems to be the more important PLA_2_ isoform in airway inflammation, especially caused by house dust mite allergens. Der p1 has protease activity and can actively degrade and penetrate the epithelium barrier in airways. Moreover stimulation of sPLA_2_X production whose activity is connected with further cysteinyl leukotrienes synthesis may sustain inflammatory process. This phenomenon might be supported additionally by decreased synthesis of cPLA_2_ and subsequent diminished PGE_2_ synthesis, which in respiratory track may also play protective role. Fel d1 seems to act rather by decreasing the cPLA_2_ expression than induction of sPLA_2_X. Further studies focusing on expression of different PLA_2_ isoforms in different timepoints after inflammatory stimulus exposition should be analyzed to better understand the molecular mechanism of allergic inflammation.

## Figures and Tables

**Table 1:** Characteristics of patients with bronchial asthma and healthy subjects.

	Asthmatic patients	Healthy volunteers
*N*	7	7
Age (years)	48 (39–72)	36 (24–41)
Women/men	5/2	5/2
Smokers/nonsmokers	1/7	0/7
Inhaled GCS (*μ*g/day)^a^	1508 (960–1600)	0
Systemic GCS (mg/day)^b^	18,8 (7–20)	0
FEV1 (%)	74.6	N/A
Allergic to Der p1	6/7	No
Allergic to Fel d1	3/7	No

^a^Doses were calculated as budesonide equivalents. ^b^Doses were calculated as prednisone equivalents.
